# Epigenetic Mechanisms of Vitamin D in the Aging Process: A Narrative Review

**DOI:** 10.3390/ijms27156578

**Published:** 2026-07-24

**Authors:** Yi Guo, Yizhen Yan, Li Zhao, Shanshan Mao

**Affiliations:** 1 Beijing Key Laboratory of Sports Performance and Skill Assessment, Beijing Sport University, Beijing 100084, China; yi___guo@163.com (Y.G.); zhaolispring@126.com (L.Z.); 2 School of Sports Medicine and Rehabilitation, Beijing Sport University, Beijing 100084, China; yanyiz@126.com

**Keywords:** vitamin D, aging, epigenetic mechanisms, VDR, DNA methylation, histone modification

## Abstract

Aging is driven by progressive epigenetic alterations—DNA methylation drift, aberrant histone modifications, chromatin remodeling, and non-coding RNA dysregulation. Vitamin D, acting through its nuclear receptor vitamin D receptor (VDR), modulates the epigenetic landscape to potentially counteract these age-related changes. This review first describes age-related epigenetic alterations, then outlines vitamin D signaling and its interface with the epigenetic machinery. Next, tissue-specific epigenetic actions of vitamin D in the immune, musculoskeletal, and nervous systems are discussed. Finally, clinical trial evidence is examined, interindividual variability is highlighted, and future research directions are proposed. However, large randomized controlled trials (RCTs) consistently show limited benefits of vitamin D monotherapy, with measurable anti-aging effects observed when combined with exercise and nutritional interventions. Its efficacy is constrained by interindividual variability, J-shaped dose–response, and tissue-specific barriers. For deficient individuals (serum 25-hydroxyvitamin D (25(OH)D) < 50 nmol/L), guided supplementation—typically 800–2000 international units (IU)/day—is warranted to achieve tentative target serum concentrations of 75–125 nmol/L, the range linked to favorable epigenetic and immune effects. For those already sufficient (e.g., serum 25(OH)D ≥ 50 nmol/L), indiscriminate supplementation without biochemical indication is not supported. Therefore, promoting healthy aging through vitamin D requires serum-monitored, individually titrated, and multimodal regimens, with supplementation reserved primarily for documented deficiency and integrated with lifestyle interventions.

## 1. Introduction

Aging itself is a complex biological process driven by progressive epigenetic alterations—DNA methylation drift, aberrant histone modifications, chromatin remodeling, and non-coding RNA dysregulation [1,2]. These changes collectively lead to genomic instability, stem cell exhaustion, mitochondrial dysfunction, and chronic inflammation—the hallmarks of aging [3,4]. Importantly, many of these epigenetic changes are reversible and can be modulated by environmental and nutritional factors [5,6]. Among these, vitamin D has emerged as a potent epigenetic regulator through its nuclear receptor vitamin D receptor (VDR) [7,8,9]. VDR is a transcription factor that recruits pioneer factors such as PU.1 and CCAAT/enhancer-binding protein α (C/EBPα) to open chromatin and direct cell-type-specific epigenetic programming [9,10,11]. Through these mechanisms, vitamin D influences gene expression networks controlling immune function, muscle maintenance, bone metabolism, and neuroprotection—all of which deteriorate with age [8,12].

Before proceeding, it is important to clarify three distinct but related concepts that are frequently conflated in the vitamin D literature [8]. Serum 25-hydroxyvitamin D (25(OH)D) is the primary biomarker of vitamin D status, reflecting cumulative supply from cutaneous synthesis, dietary intake, and oral supplementation; it is the standard measure used to define deficiency, insufficiency, and sufficiency [13]. 1,25-Dihydroxyvitamin D_3_ (1,25(OH)_2_D_3_) is the biologically active hormone that binds to VDR, but its circulating levels are tightly regulated by renal CYP27B1 activity and calcium–phosphate homeostasis; thus, it is not a reliable indicator of overall vitamin D status [8]. Finally, oral supplementation dose (measured in IU/day) does not directly equate to serum 25(OH)D concentration, as the conversion between dose and serum level is modulated by individual differences in absorption, metabolism, baseline status, BMI, skin pigmentation, and genetic factors [14,15]. Throughout this review, we use “serum 25(OH)D” when referring to the biomarker of status, “supplementation dose” when referring to intervention parameters, and “vitamin D” as a general term for the nutrient and its metabolites.

Notably, vitamin D deficiency is highly prevalent among older adults worldwide (affecting approximately 20–40% of this population), and this deficiency is associated with increased risks of age-related diseases and accelerated functional decline [13,16]. Maintaining serum 25(OH)D levels within a suggested range (e.g., 75–125 nmol/L) has been proposed by some studies as a potentially actionable approach to promote healthy aging [8,17]. However, large randomized controlled trials have yielded mixed results regarding the benefits of vitamin D supplementation alone, with some studies showing little to no effect on age-related outcomes [18]. This apparent discrepancy—between observational associations and trial findings—can be reconciled by considering the nature of age-related epigenetic alterations. Such alterations (e.g., DNA methylation drift, histone modification imbalances) are long-term cumulative and highly individualized, potentially leaving “epigenetic scars” that short-term, one-size-fits-all supplementation may fail to erase. Vitamin D acts as an epigenetic modulator via its nuclear receptor VDR [8], but reversing established epigenetic changes likely requires personalized strategies tailored to an individual’s baseline epigenetic landscape.

Therefore, understanding the interface between vitamin D signaling and the epigenetic machinery is critical to explain interindividual variability in vitamin D responsiveness. This review focuses on the epigenetic mechanisms by which vitamin D regulates aging, with an emphasis on translating these findings into actionable strategies. In this review, we synthesize current evidence on VDR-mediated epigenetic mechanisms and discuss their implications for personalized strategies to promote healthy aging.

## 2. Methods

This narrative review was based on a comprehensive literature search of the PubMed, Scopus, and Web of Science databases. We included peer-reviewed publications from the databases’ inception through July 2026, focusing on research related to vitamin D, epigenetic regulation, and aging.

The search strategy was constructed using combinations of the following core keywords: “vitamin D”, “vitamin D receptor (VDR)”, “aging”, “epigenetic mechanisms”, “DNA methylation”, “histone modification”, “chromatin remodeling”, “non-coding RNAs”, “immunosenescence”, “sarcopenia”, and “neurodegenerative diseases”. Boolean operators (AND/OR) were used to optimize the accuracy and comprehensiveness of the search. Inclusion criteria included:

- Original research articles (in vitro, in vivo, clinical trials) and high-quality narrative/systematic reviews;
- Studies published in English;
- Studies focusing on the epigenetic regulatory effects of vitamin D in the aging process and age-related diseases;
- Studies reporting molecular mechanisms related to VDR signaling, DNA methylation, histone modifications, chromatin remodeling, or non-coding RNAs.

Exclusion criteria included:

- Non-English publications;
- Conference abstracts, case reports, letters, and editorials without full-text data;
- Studies irrelevant to the core theme of vitamin D-mediated epigenetic regulation in aging.

The literature screening process was conducted in two steps: initial screening of titles and abstracts, followed by full-text evaluation of potentially eligible studies. The final included studies were selected based on their scientific quality, novelty, and direct relevance to the epigenetic mechanisms of vitamin D in aging.

**Figure 1:**
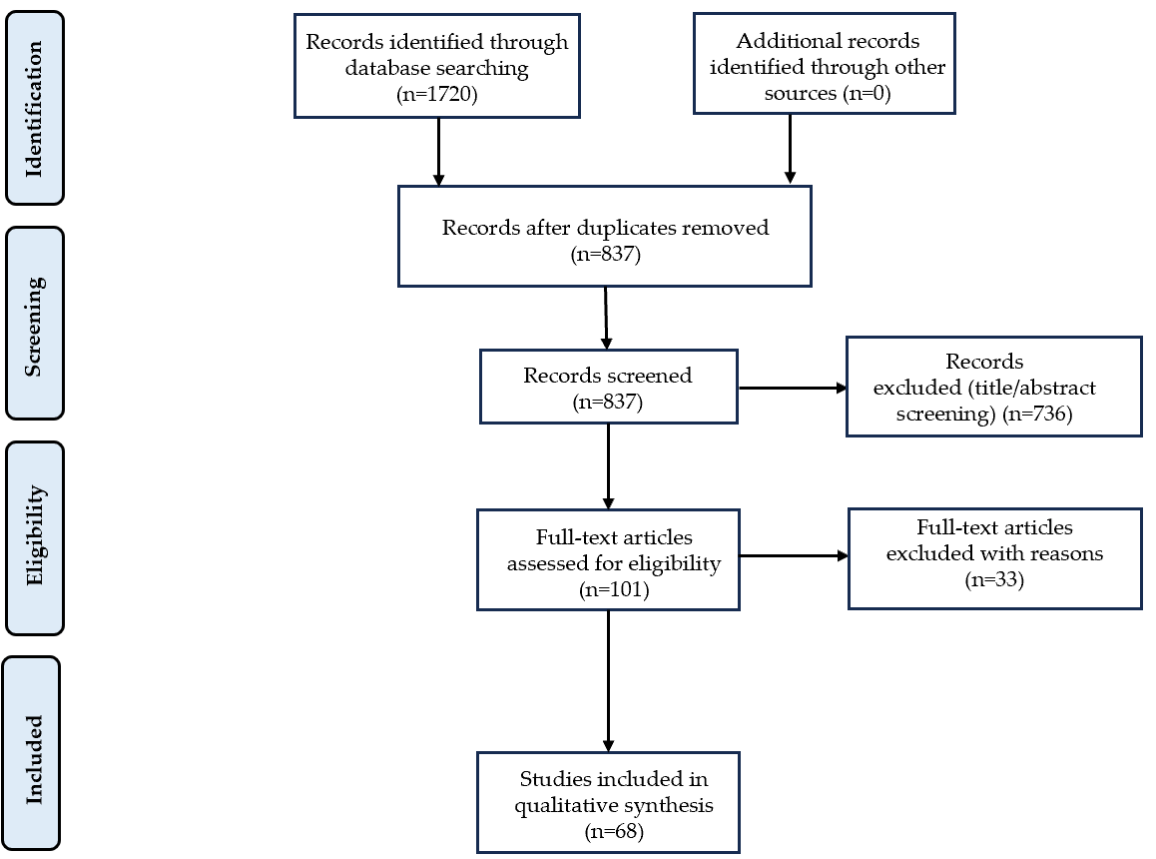
Preferred Reporting Items for Systematic Reviews and Meta-Analyses (PRISMA) 2020 flow diagram of the literature search and study selection process for this narrative review.

A total of 1720 records were initially retrieved from PubMed, Scopus, and Web of Science. After removing duplicates, 837 records remained for title and abstract screening. Following the pre-specified exclusion criteria, 101 articles were retrieved for full-text evaluation. Finally, 68 studies met all inclusion criteria and were included in the qualitative synthesis of this narrative review. The detailed literature search and study selection process is illustrated in the Preferred Reporting Items for Systematic Reviews and Meta-Analyses (PRISMA) 2020 flow diagram (Figure 1).

The screening process was conducted in two stages: (A) title and abstract screening against the inclusion criteria, and (B) full-text assessment of potentially eligible articles. The primary reasons for exclusion at the full-text stage were: (a) non-original research (e.g., editorials, case reports), (b) lack of direct relevance to vitamin D-mediated epigenetic mechanisms in aging, and (c) insufficient mechanistic detail.

## 3. Epigenetic Alterations During Aging

Aging is accompanied by multiple epigenetic alterations. First, global DNA hypomethylation—particularly at repetitive elements such as long interspersed nuclear element 1 (LINE-1), Alu sequences, and satellite repeats—coexists with CpG island hypermethylation at promoters of tumor suppressor genes (e.g., *p16INK4a*, *ESR1*, *RUNX2*), leading to genomic instability and silencing of protective genes [19,20]; second, histone modifications become aberrant, including loss of heterochromatin-associated repressive marks such as H3K9me3 and H3K27me3, and abnormal accumulation of active marks like H3K27ac at pro-inflammatory gene loci, which promotes chronic inflammation and transcriptional dysregulation [19]; third, chromatin remodeling complex activity declines with age, resulting in chromatin compaction, reduced accessibility of transcription factors to their target sites, and disrupted enhancer–promoter looping, thereby impairing precise gene regulation [20]; and fourth, non-coding RNAs—particularly microRNAs (miRNAs)—become dysregulated; for example, pro-aging miRNAs such as the miR-34 family and miR-21 are upregulated, while anti-aging miRNAs including the miR-29 family and miR-215 are downregulated, contributing to cellular senescence and impaired tissue repair [5,21].

Together, these interconnected epigenetic changes drive genomic instability, transcriptional noise, stem cell exhaustion, and chronic low-grade inflammation—all of which are recognized hallmarks of aging [19,21]. The cumulative effect of these alterations accelerates functional decline across multiple organ systems and increases susceptibility to age-related diseases, including neurodegeneration, sarcopenia, and immunosenescence.

## 4. Vitamin D Signaling and Its Interface with Epigenetic Machinery

Most mechanistic insights into VDR-mediated epigenetic regulation described in this section are derived from in vitro cell models and preclinical animal studies. Direct in vivo evidence from human tissues remains relatively limited, and translational validation is required to confirm the conservation of these mechanisms in human aging.

### 4.1. VDR Signaling: From DNA Binding to Chromatin Regulation

The VDR belongs to the nuclear receptor superfamily and consists of a DNA-binding domain (DBD) and a ligand-binding domain (LBD) [22]. Upon binding of the active metabolite 1,25(OH)_2_D_3_ to the LBD, VDR heterodimerizes with the retinoid X receptor (RXR) and recognizes vitamin D response elements (VDREs) in the genome, typically as direct repeats with a three-base-pair spacing (DR3-type) [23].

VDR requires pioneer factors to access closed chromatin. In immune cells, PU.1 and C/EBPα are key pioneer factors: they bind nucleosomal DNA, recruit chromatin remodeling complexes and histone acetyltransferases, increase chromatin accessibility, and thereby create open regions for VDR binding [9,24].

High-throughput technologies such as chromatin immunoprecipitation sequencing (ChIP-seq) and assay for transposase-accessible chromatin using sequencing (ATAC-seq) have revealed the genome-wide binding pattern of VDR. VDR binding sites are tissue-specific, located predominantly in enhancer regions, and co-localize with active epigenetic marks (H3K4me1, H3K27ac) [8,25]. Vitamin D stimulation induces dynamic changes in thousands of VDR binding sites and global remodeling of chromatin accessibility [25].

VDR regulates distal target genes through enhancer–promoter looping. Three-dimensional genomics techniques such as high-throughput chromosome conformation capture (Hi-C) and chromatin interaction analysis by paired-end tag sequencing (ChIA-PET) have shown that VDR-bound enhancers physically contact target gene promoters via DNA loops [26]. Vitamin D can strengthen existing looping structures or induce new ones, thereby facilitating transcriptional activation. Age-related alterations in three-dimensional genome architecture may impair this regulation [25].

Vitamin D exerts genome-wide epigenetic and transcriptional effects via VDR. As its core mechanism, VDR recruits histone acetyltransferase (HAT) co-activators (e.g., p300/CBP) to deposit H3K27ac at enhancers and drive chromatin opening; it also modulates DNA methylation via DNA methyltransferases (DNMTs) and ten-eleven translocation methylcytosine dioxygenases (TETs) and interacts with chromatin remodeling complexes [27]. Transcriptionally, vitamin D regulates genes involved in immune function, bone metabolism, cell cycle, and antioxidant defense, and chromatin accessibility changes are strongly correlated with target gene upregulation [8,28]. Multi-omics integration reveals that vitamin D-induced chromatin opening correlates strongly with gene upregulation [8]. Of note, aging-related alterations in chromatin rigidity and reduced VDR expression may attenuate the efficiency of this regulatory network, although this remains an area of active investigation [25].

### 4.2. Epigenetic Reprogramming by Vitamin D

#### 4.2.1. Regulation of DNA Methylation by Vitamin D

Vitamin D regulates DNA methylation via VDR. VDR can bind to the promoters of DNMTs and TET enzymes, modulating their expression: for example, upregulating *TET2* to promote 5-hydroxymethylcytosine (5-hmC) formation and inhibiting *DNMT1* to reduce global methylation [27,29]. The Vitamin D Supplementation, Omega-3 Fatty Acid Supplementation, and Strength-Training Exercise Program (DO-HEALTH) trial showed that vitamin D combined with exercise and nutritional interventions significantly slowed epigenetic clocks such as PhenoAge and GrimAge2 (equivalent to 2.9–3.8 months of biological age deceleration) [30]. Moreover, vitamin D is associated with maintenance of telomeric methylation and slower telomere attrition [31,32,33].

Vitamin D regulates CpG island methylation of specific genes relevant to aging [27,34]. For example, vitamin D deficiency has been associated with hypermethylation of the estrogen receptor gene (*ESR1*) promoter in aged tissues, leading to reduced *ESR1* expression and accelerated cellular senescence [35]. Conversely, vitamin D repletion can reverse age-related hypermethylation of the tumor suppressor gene *p16INK4a*, thereby preserving cell cycle control and preventing premature senescence [34]. Mechanistically, VDR directly binds to the promoter regions of *DNMT1* and *DNMT3B*, suppressing their transcription and reducing global DNA methylation levels [27]. In vitamin D deficient states, elevated DNMT activity contributes to genome-wide hypomethylation of repetitive elements (e.g., LINE-1, Alu), promoting genomic instability—a hallmark of aging [34]. Thus, vitamin D acts as a potential regulator of the methylome, partially counteracting aberrant hypermethylation of protective genes and harmful hypomethylation of repetitive DNA [27,35,36]. Notably, this regulatory effect is context-dependent, restricted by baseline epigenetic status and VDR expression levels [29].

#### 4.2.2. Regulation of Histone Modifications by Vitamin D

Beyond the canonical acetylation pathway outlined in Section 4.1, VDR exerts bidirectional histone regulation to counteract age-related dysregulation: it inhibits histone acetyltransferase (HAT) 1 to preserve repressive marks such as H3K9me2, and suppresses Polycomb repressive complex 2 (PRC2) activity to normalize H3K27me3 levels at cell cycle genes [27]. In aged cells, this restores heterochromatin stability and dampens excessive pro-inflammatory transcription [34].

Vitamin D also modulates specific histone methyltransferases and demethylases [37]. Emerging evidence from in vitro studies suggests that VDR activation may influence the expression of histone methyltransferases potentially involving enzymes including SETD7 (a H3K4 methyltransferase) and demethylases such as KDM6B (a H3K27 demethylase) [38], potentially modulating H3K4me1 deposition at enhancers and H3K27me3-mediated repression [38,39]. Additionally, in vitro studies have suggested that vitamin D may modulate EZH2 activity in aged immune cells, potentially affecting H3K27me3 deposition at cytolytic gene promoters [40,41]. These findings suggest that vitamin D may fine-tune the histone code in the aging epigenome under favorable conditions.

#### 4.2.3. Regulation of Chromatin Accessibility and 3D Genome by Vitamin D

Vitamin D regulates chromatin accessibility and three-dimensional genome organization via VDR [10]. VDR interacts with remodeling complexes such as switch/sucrose non-fermentable (SWI/SNF) and Mediator, increasing open chromatin regions (as confirmed by ATAC-seq) [24,25,26]. Hi-C analyses in cell models have suggested that vitamin D may induce new enhancer-promoter loops and influence topologically associating domain (TAD) boundary stability, potentially counteracting age-related disruption of three-dimensional architecture [42]. However, direct evidence for TAD stabilization by vitamin D in aging tissues remains limited, and most data are derived from in vitro systems [43]. These actions collectively contribute to maintaining the dynamic balance of chromatin structure in aging cells [44,45].

#### 4.2.4. Regulation of Non-Coding RNAs (miRNAs) by Vitamin D

Vitamin D regulates miRNA expression via VDR [46]. VDR can directly bind to the promoters of miRNA genes (e.g., miR-106b, miR-181a), and their expression increases upon 1,25(OH)_2_D_3_ stimulation. Vitamin D suppresses pro-aging miR-34 family and miR-21, while promoting anti-aging miR-29 family and miR-215 [47]. ATAC-seq and Hi-C reveal that vitamin D increases chromatin accessibility and enhancer–promoter looping at miRNA gene loci, enabling precise regulation [48]. In aged immune cells, vitamin D inhibits miR-34a to reduce *IL-6* secretion and promotes miR-29b to enhance tissue repair [47,49].

Beyond miRNAs, vitamin D has been reported to modulate the expression of long non-coding RNAs (lncRNAs) linked to senescence and inflammation, including H19 and MALAT1, although most evidence is derived from cancer models or in vitro systems [50]. Emerging evidence also suggests that vitamin D may modulate circular RNAs (circRNAs) linked to aging, although this area remains largely unexplored [8]. These emerging data suggest that vitamin D orchestrates a complex non-coding RNA network, fine-tuning both transcriptional and post-transcriptional programs during aging. However, vitamin D-mediated miRNA regulation exhibits cell-type specificity and cannot override established aging-related non-coding RNA dysregulation [47].

**Figure 2:**
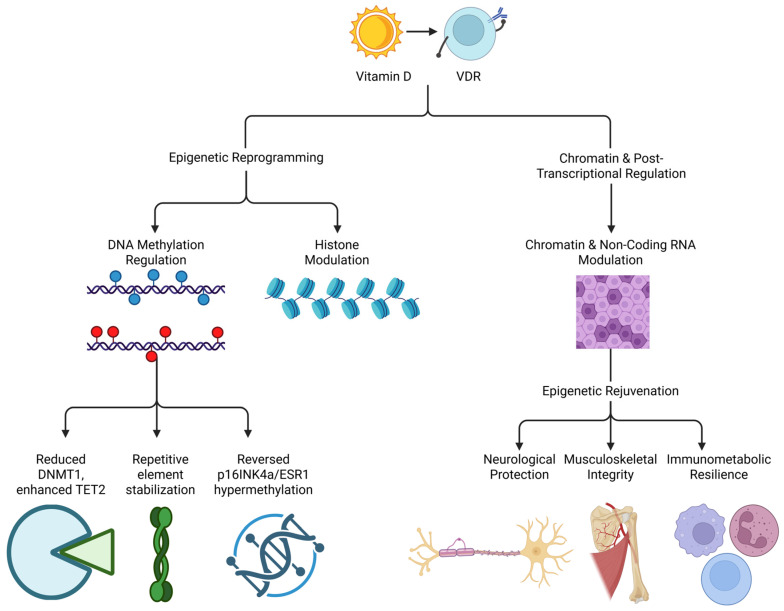
Vitamin D-mediated epigenetic mechanisms counteract aging. Vitamin D/VDR signaling modulates DNA methylation, histone modifications, chromatin structure, and non-coding RNAs to promote epigenetic rejuvenation. This enhances genomic stability and reduces inflammation, supporting multi-tissue resilience against age-related decline. Created with BioRender.com.

Collectively, vitamin D modulates epigenetic machinery via VDR, but these regulatory processes are not universal—their efficacy is constrained by tissue-specific VDR availability and age-related chromatin rigidity [51] (Figure 2).

## 5. Epigenetic Mechanisms by Which Vitamin D Regulates Aging in Key Organ Systems

Vitamin D exerts tissue-specific epigenetic regulation via VDR across the immune, musculoskeletal, and nervous systems, involving DNA methylation, histone modifications, chromatin remodeling, and non-coding RNA networks. These mechanisms collectively contribute to delaying age-related functional decline and provide a theoretical basis for precision anti-aging interventions [8]. Table 1 summarizes representative core studies supporting VDR-mediated epigenetic regulation in aging and age-related conditions, with explicit annotation of study design, experimental model, and evidence level to support critical evaluation of translational potential.

### 5.1. Immune System

Preclinical studies and observational evidence suggest that vitamin D modulates immune aging through epigenetic pathways, as detailed below.

Immunosenescence is characterized by myeloid skewing of hematopoietic stem cells (HSCs) and chronic inflammation [61,62]. Vitamin D, via VDR and pioneer factors PU.1 and C/EBPα, regulates epigenetic programming of HSCs, thereby maintaining open chromatin regions and preventing premature HSC exhaustion [9,10,37,63,64]. VDR directly targets *IL-6* and *TNF-α* promoters to reduce H3K27ac and suppress SASP factor production [27,65,66,67,68]. Vitamin D also enhances the epigenetic memory of trained immunity, improving vaccine responses in the elderly. Vitamin D deficiency upregulates DNMT3A, causing hypermethylation of HOX genes and accelerating immunosenescence [69].

Vitamin D also epigenetically suppresses the senescence-associated secretory phenotype (SASP) by targeting chemokine genes [52,70,71]. Similarly, VDR reduces H3K27ac at *CXCL8* and *CCL2* loci via HDAC2 to restrict chemokine secretion and chronic low-grade inflammation [72]. Furthermore, vitamin D influences T cell epigenetic aging. In aged CD8+ T cells, VDR activation enhances *TET2* expression, promoting DNA demethylation at perforin and granzyme B loci and restoring cytotoxic function. Conversely, vitamin D deficiency accelerates T cell exhaustion, characterized by increased methylation of *PDCD1* (encoding PD-1) and reduced effector function [34,73]. Thus, adequate vitamin D status can help preserve both innate and adaptive immune competence by maintaining a youthful epigenetic landscape in immune cells [74]. However, it is important to note that large-scale RCTs have not consistently demonstrated that vitamin D supplementation alone translates these mechanistic effects into tangible clinical benefits, such as reduced infection rates or improved vaccine responses in the general elderly population [18,75].

Despite the above epigenetic actions, the translational impact of vitamin D on immunosenescence has substantial limitations. First, VDR polymorphisms cause marked interindividual variability: FokI FF carriers exhibit 1.7-fold higher responsiveness than ff carriers, while BsmI BB individuals require serum 25(OH)D levels of 75–87.5 nmol/L to achieve adequate immunomodulation [29]. Second, the dose–response follows a J-shaped curve: 2000 IU/day effectively reduces *IL-6*, but sustained daily doses > 4000 IU/day may provoke calcium overload via TRPV6 channels, leading to immunosuppression or paradoxical hyperinflammation [54]. Third, immune cell-specific expression of CYP27B1 differs: Neutrophils lack significant CYP27B1 and cannot produce active vitamin D locally, whereas macrophages, dendritic cells and T cells can, making the net effect cell-type-dependent [53]. Fourth, epigenetic benefits require prolonged supplementation and are reversible: The VITamin D and OmegA-3 TriaL (VITAL) trial showed that four years of treatment were needed to observe significant slowing of telomere attrition, and these effects disappear upon discontinuation, necessitating lifelong maintenance [75].

Therefore, vitamin D alone is unlikely to reverse established immunosenescence; its use should be guided by VDR genotyping, J-shaped dose titration, cell-type-specific targeting, and lifelong maintenance.

### 5.2. Musculoskeletal System

Preclinical studies and observational evidence suggest that vitamin D modulates Musculoskeletal aging through epigenetic pathways, as detailed below.

#### 5.2.1. Skeletal Muscle

Sarcopenia is accompanied by progressive epigenetic alterations in skeletal muscle and satellite cells, including hypermethylation of the *MyoD* and *myogenin* promoters and reduced H3K27 acetylation, which may impair myogenic differentiation and regenerative capacity [61,76,77,78]. Through VDR-mediated transcriptional regulation, vitamin D can interact with epigenetic machinery and muscle-specific transcription factors to modulate genes involved in muscle growth, differentiation, and metabolism. VDR may cooperate with the pioneer transcription factor C/EBPα and promote anabolic signaling by facilitating H3K27ac deposition at loci associated with *IGF-1* and follistatin [9]. In vitro studies further suggest that vitamin D may suppress catabolic signaling involving myostatin and regulate mitochondrial biogenesis through the *PGC-1α* network, although direct evidence for vitamin D-induced demethylation of the *MSTN* promoter in human skeletal muscle remains limited [12,55,79,80,81,82,83]. Vitamin D may also regulate non-coding RNAs, including miR-21 and miR-146a, thereby reducing mitochondrial dysfunction and apoptosis. Collectively, these findings provide a mechanistic basis for the potential role of vitamin D in preserving skeletal muscle function during aging.

Clinically, vitamin D deficiency is highly prevalent among older adults with sarcopenia, and lower serum 25(OH)D concentrations are associated with reduced muscle mass, grip strength, gait speed, and increased fall risk [12,16]. Nevertheless, evidence from randomized controlled trials indicates that vitamin D supplementation alone generally produces modest and inconsistent improvements in muscle mass, strength, and physical performance. The limited effects of monotherapy are particularly evident in community-dwelling older adults without marked vitamin D deficiency, in whom additional supplementation has not consistently improved musculoskeletal outcomes [84]. These findings suggest that correction of deficiency may be beneficial, but vitamin D alone is unlikely to provide a sufficient stimulus to reverse the multifactorial processes underlying established sarcopenia.

More consistent benefits have been reported when vitamin D supplementation is incorporated into multimodal interventions. Meta-analyses of randomized controlled trials suggest that vitamin D supplementation, commonly at 800–2000 IU/day, combined with resistance training may improve muscle strength and reduce fall risk compared with exercise alone or usual care [85,86]. Similarly, the DO-HEALTH trial reported that a multicomponent intervention involving vitamin D, exercise, and omega-3 fatty acids was associated with modest slowing of epigenetic aging, with estimated reductions of approximately 2.9–3.8 months in selected epigenetic clocks [12,80,81,82,83]. While statistically significant, this effect size is clinically modest and its relevance to individual healthspan remains to be determined. Exercise may enhance skeletal muscle VDR expression and chromatin accessibility, whereas vitamin D may help stabilize transcriptionally active chromatin and support anabolic and mitochondrial pathways. These complementary mechanisms may explain why combined interventions produce more measurable and consistent effects than vitamin D monotherapy.

The response to vitamin D is further modified by baseline vitamin D status, tissue-specific metabolism, dose, sex, genetic background, and metabolic health. Obesity and high-fat dietary conditions may suppress local CYP27B1 activity, reduce tissue-specific production of 1,25(OH)_2_D_3_, and attenuate vitamin D signaling in skeletal muscle and bone [87]. Sex-related differences and VDR polymorphisms may also influence treatment response; for example, the DO-HEALTH trial identified a significant sex-by-vitamin D interaction for lumbar spine bone mineral density, whereas carriers of different VDR BsmI genotypes may respond differently to supplementation [51,56]. The proposed J-shaped relationship primarily applies to circulating serum 25(OH)D concentrations rather than to a universal oral-dose threshold. Moderate supplementation may support musculoskeletal health in deficient individuals, whereas prolonged high-dose supplementation exceeding 4000 IU/day has been associated with impaired balance or postural control in some studies [14,88]. Therefore, supplementation should be individualized according to serum 25(OH)D concentrations and clinical characteristics rather than based on a fixed dose alone.

Overall, vitamin D has biologically plausible and potentially beneficial effects on skeletal muscle aging, particularly in individuals with vitamin D deficiency. However, vitamin D supplementation alone has limited capacity to prevent or reverse sarcopenia because muscle aging is driven by multiple interacting anabolic, inflammatory, metabolic, and epigenetic disturbances. Vitamin D should therefore be incorporated into a serum-monitored and individually titrated multimodal strategy that includes resistance exercise, adequate protein intake, and, where appropriate, omega-3 fatty acids. In this context, vitamin D functions as a supportive component that may enhance the effectiveness of established exercise and nutritional interventions rather than as an independent treatment for sarcopenia.

#### 5.2.2. Bone and Cartilage

In the context of osteoporosis and osteoarthritis, which are major age-related bone disorders, aberrant epigenetic patterns have been detected in key osteogenic genes. It is important to note that findings from these disease models reflect pathological states and may not directly represent physiological aging processes. In these disease states, *RUNX2* and *SP7* promoters are hypermethylated, while the RANKL enhancer shows increased H3K27ac [89]. VDR mediates bidirectional bone remodeling via histone modification: it reduces H3K27ac at the RANKL enhancer to inhibit osteoclastogenesis, and increases H3K9ac at the *BGLAP* promoter to promote bone matrix deposition [27,90]. In mesenchymal stem cell models, vitamin D may restore the methylation pattern of *SFRP1* and *DKK1*, promoting osteogenesis [57]. In cartilage models, vitamin D has been reported to modulate the expression of *SOX9* and *COL2A1*, key regulators of chondrocyte function, potentially through epigenetic mechanisms such as DNA methylation and histone modifications [57].

Vitamin D also regulates chondrocyte hypertrophy, a critical step in osteoarthritis progression. VDR activation suppresses the hypermethylation of the *RUNX2* promoter in chondrocytes, preventing premature hypertrophy and matrix degradation [91]. Furthermore, vitamin D modulates the Wnt/β-catenin pathway through epigenetic control. In aged bone, vitamin D reduces methylation of the *DKK1* (a Wnt antagonist) promoter, restoring *DKK1* expression and limiting aberrant Wnt signaling, which otherwise drives osteoclastogenesis [92,93,94]. Additionally, vitamin D enhances H3K9ac at the SOST (sclerostin) promoter, inhibiting sclerostin production and promoting bone formation [95].

However, the epigenetic anti-osteoporotic effects of vitamin D are often overestimated. Large RCTs showed that vitamin D supplementation alone (without calcium) does not reduce fracture risk or significantly improve bone mineral density in community-dwelling older adults with baseline 25(OH)D levels above 50 nmol/L [96,97]. Of note, the 50 nmol/L threshold used in these trials reflects bone-specific guidelines, which differ from the 75–125 nmol/L range discussed in this review for epigenetic and immune outcomes. This highlights that the ‘optimal’ serum 25(OH)D concentration is outcome-dependent, and no single cutoff applies universally. Therefore, correcting severe deficiency is critical, but supplementation beyond sufficiency confers little additional epigenetic benefit for bone and cartilage.

### 5.3. Nervous System

Preclinical studies and observational evidence suggest that vitamin D modulates neural aging through epigenetic pathways, as detailed below.

In Alzheimer’s disease and other neurodegenerative conditions, which increase in prevalence with advancing age, disease-specific epigenetic dysregulation of amyloid and tau regulatory genes has been identified. The following mechanisms are primarily derived from disease-specific models; their relevance to physiological brain aging requires further investigation. In these pathological states, *APP* and *BACE1* promoters have been reported to be hypomethylated, *MAPT* shows altered methylation patterns, and H3K9me3 is diminished in certain brain regions. VDR binds these gene promoters, recruits DNMTs to increase methylation, and suppresses Aβ and tau production [98,99]. Vitamin D also increases H3K27ac at *BDNF* and *NGF* promoters, promoting neurotrophic factor expression.

In Parkinson’s disease, VDR and its metabolic enzymes are widely expressed in the substantia nigra [100], and vitamin D has been shown to upregulate *BDNF* and *GDNF* expression while suppressing microglial activation and neuroinflammation [101]. Cell and animal studies have further revealed that vitamin D may inhibit α-synuclein aggregation through calbindin-D28k-mediated calcium homeostasis [59], and VDR polymorphisms such as FokI have been associated with PD susceptibility [58]. As with Huntington’s disease (HD) and amyotrophic lateral sclerosis (ALS), these PD-specific findings derive from disease models and do not represent physiological biology. Clinically, meta-analyses suggest that vitamin D supplementation may improve daily activity endurance (e.g., 6-min walking test), although effects on UPDRS-III motor scores remain inconclusive [102], and deficiency has been linked to non-motor symptoms including mood and cognitive disturbances [103]. The following observations in HD and ALS are similarly derived from disease-specific contexts and should not be equated with mechanisms of physiological brain aging.

In Huntington’s disease, all available mechanistic evidence originates solely from preclinical pathological models and does not capture physiological brain aging. Preclinical studies in the 3-nitropropionic acid-induced mouse model of HD, which recapitulates striatal pathology rather than physiological aging, have shown that vitamin D_3_ supplementation restores α7 nicotinic acetylcholine receptor expression and reduces acetylcholinesterase activity, thereby rescuing cholinergic signaling and alleviating motor and cognitive dysfunction [60]. Vitamin D also downregulates T-cell receptor β subunit expression, suppresses microglial activation, and upregulates *BDNF* through VDR binding to vitamin D response elements, providing neurotrophic support for degenerating striatal neurons [58,60]. Clinically, vitamin D deficiency is highly prevalent in HD patients, affecting up to 89% of individuals, and correlates with impaired motor function and increased fall risk [104]. However, these findings are derived from patients with established neurodegeneration and cannot be extrapolated to physiological brain aging or used to support routine vitamin D supplementation for neuroprotection in healthy older adults. In amyotrophic lateral sclerosis, the relationship with vitamin D remains incompletely characterized, with most evidence derived from observational studies and animal models, and large-scale clinical trials are lacking [102]. As with HD, findings from ALS models reflect disease-specific pathology rather than normative age-related changes, and their translational relevance to healthy aging remains uncertain.

**Figure 3:**
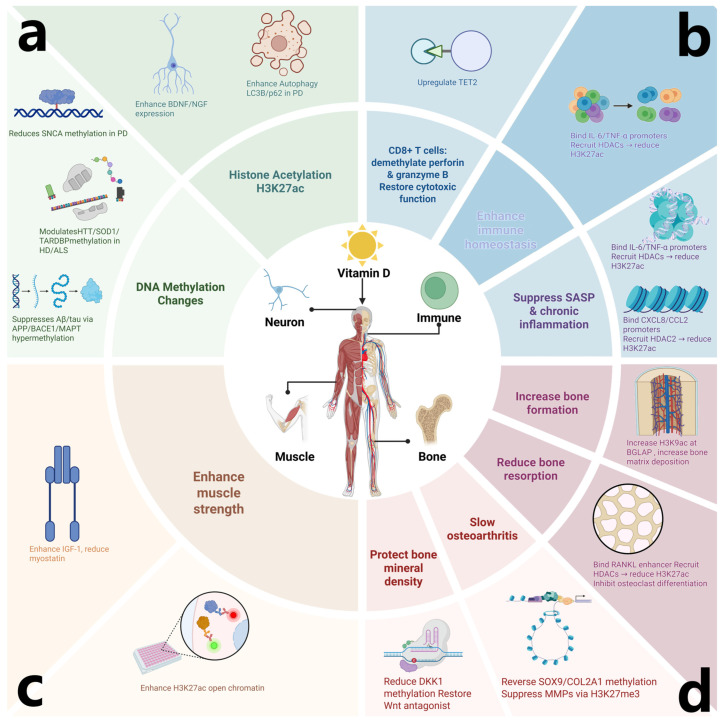
Tissue-specific epigenetic regulation by vitamin D/VDR signaling counteracts aging hallmarks across four tissue systems, labeled (**a**) to (**d**): (**a**) Neural: VDR induces *APP/BACE1* hypermethylation, elevates H3K27ac at the *BDNF* promoter, and modulates *SNCA/HTT/TARDBP* methylation in neurodegenerative contexts. (**b**) Immune: VDR suppresses SASP via HDAC-mediated H3K27ac reduction at cytokine loci, and restores CD8+ T cell cytotoxicity by upregulating *TET2*. (**c**) Skeletal muscle: VDR enhances H3K27ac-dependent chromatin opening to upregulate *IGF-1* and repress *MSTN*, promoting muscle strength. (**d**) Bone/cartilage: VDR reduces RANKL enhancer H3K27ac to inhibit osteoclastogenesis, restores *SOX9*/*COL2A1* methylation, and modulates *DKK1*/*BGLAP* to maintain bone homeostasis. Within each system, color depth corresponds to the strength of supporting evidence, with darker shades indicating stronger experimental support. Created with BioRender.com.

The application of vitamin D-mediated epigenetic strategies, including their clinical translation, faces major hurdles [105]. First, the blood–brain barrier severely restricts the entry of the circulating precursor, 25-hydroxyvitamin D (25(OH)D). Brain concentrations of total 25(OH)D are reported to be only 10–30% of serum levels [106]. It is important to distinguish this from the locally activated hormone, 1,25-dihydroxyvitamin D_3_ (1,25(OH)_2_D_3_), whose concentration within the brain is tightly regulated by local CYP27B1 activity and may not directly reflect serum levels. The clinical significance of these differential concentrations, particularly regarding their impact on VDR-mediated epigenetic effects in the brain, remains largely unknown and is a critical area for future research. Second, active vitamin D in the brain depends largely on local CYP27B1 expression: This is restricted to neurons and glia and is regulated by inflammatory state, making central effects indirect and variable [53]. Third, no large RCT has shown that vitamin D alone in any neurodegenerative disease: To date, no large RCT has shown that vitamin D alone improves cognitive decline, slows Alzheimer’s disease progression, or alters clinical trajectories in Parkinson’s, Huntington’s or amyotrophic lateral sclerosis [107]. Fourth, potential neurotoxicity has been reported in the context of long-term high-dose oral supplementation. For example, one study noted that 2000 IU/day may improve certain memory parameters, whereas daily doses ≥ 4000 IU/day administered over 1 year or longer were associated with impaired reaction time and processing speed in some participants, potentially via *TRPV1*-mediated calcium overload [108]. However, these observations pertain to specific supplementation regimens and do not establish a universal serum 25(OH)D threshold for neurotoxicity, as the conversion from dose to serum concentration varies substantially across individuals due to differences in BMI, skin pigmentation, and genetic factors. Therefore, vitamin D-mediated epigenetic neuroprotection is unlikely to be effective as a standalone intervention due to BBB restriction, dependence on variable local CYP27B1, absence of large RCT evidence, and J-shaped neurotoxicity (Figure 3).

## 6. Discussion

A critical distinction must be made throughout this review: the epigenetic mechanisms described in Section 4 and Section 5 are predominantly derived from in vitro experiments and animal models. While these findings provide valuable mechanistic insights, their direct translation to human aging remains largely unproven unless confirmed by clinical studies. The following sections explicitly differentiate between mechanistic hypotheses, observational associations, and randomized trial evidence.

Vitamin D, via its nuclear receptor VDR, regulates multiple layers of the epigenetic machinery—including DNA methylation, histone modifications, chromatin remodeling, and non-coding RNAs—and thereby has the potential to exert tissue-specific anti-aging effects in the immune, musculoskeletal, and nervous systems [8,27]. The core epigenetic mechanisms identified in this review include: (a) modulating DNMTs and TETs to alter the methylation status of aging-related genes (e.g., reversing hypermethylation of *Cyclin D1*); (b) regulating histone modifications through recruitment of p300/CBP or HDACs (e.g., reducing H3K27me3 to enhance heterochromatin stability, or suppressing H3K27ac to dampen pro-inflammatory gene expression); (c) maintaining SWI/SNF-mediated chromatin accessibility; and (d) modulating miRNA networks (e.g., suppressing miR-34, upregulating miR-29) [8].

### 6.1. Clinical Evidence and Its Limitations

The epigenetic mechanisms by which vitamin D regulates aging—modulating DNA methylation, histone modifications, chromatin remodeling, and non-coding RNAs—are mechanistically well-supported by in vitro and preclinical studies. However, the translation of these findings to clinical practice is constrained by several factors: modest and inconsistent effects in large RCTs, substantial interindividual variability, a non-linear dose–response relationship, and tissue-specific barriers. The following sections critically examine the clinical evidence, sources of heterogeneity, and the rationale for multimodal strategies, before outlining future research priorities.

A major limitation is that most epigenetic mechanistic data are based on preclinical models. Human in vivo evidence of vitamin D-mediated epigenetic rejuvenation remains scarce and is largely limited to observational associations and peripheral blood biomarkers [13].

Clinically, the DO-HEALTH trial demonstrated that combined supplementation with vitamin D (2000 IU/day), omega-3 fatty acids, and a home exercise program slowed epigenetic aging clocks by 2.9–3.8 months over three years [109]. It is important to note that DO-HEALTH used a fixed oral dose (2000 IU/day) as an intervention; the observed effects cannot be directly translated to a specific serum 25(OH)D target without considering baseline status and individual metabolic variability. Similarly, the VITAL trial confirmed that four years of vitamin D supplementation significantly slowed telomere attrition. Crucially, in neither trial did vitamin D alone achieve statistical significance, underscoring that monotherapy is insufficient for reversing established epigenetic alterations. Vitamin D deficiency affects 20–40% of older adults and is consistently associated with increased risks of multiple age-related diseases [13,34,110]; however, correcting deficiency is not equivalent to reversing aging-associated epigenetic damage [8].

In this review, we propose serum 25(OH)D concentrations of 75–125 nmol/L as a tentative target for epigenetic rejuvenation and immune homeostasis [111,112]. This proposition is primarily supported by mechanistic studies demonstrating optimal VDR-mediated chromatin regulation and by observational data linking this range to favorable immune and inflammatory profiles [8,113,114]. However, we acknowledge that this target is not directly derived from large-scale RCTs, which typically use fixed dosing regimens. For instance, the DO-HEALTH trial, which observed decelerated epigenetic aging, used a fixed 2000 IU/day dose rather than a protocol targeting specific serum levels [30]. Furthermore, this proposed range is higher than the 50 nmol/L threshold established for bone health, reflecting the outcome-dependent nature of vitamin D requirements; immune and epigenetic functions may necessitate higher circulating concentrations than those required for fracture prevention. Given the lack of direct dose–response trial evidence, this range should be considered provisional and hypothesis-generating rather than a definitive clinical guideline. Mechanistic studies investigating VDR-mediated chromatin regulation and the DO-HEALTH randomized controlled trial—which observed decelerated epigenetic aging clocks under 2000 IU daily supplementation—collectively indicate that serum 25(OH)D within this bracket is required to generate measurable improvements in immunosenescence, suppression of pro-inflammatory transcription, and normalized DNA methylation landscapes [113,114].

When interpreting observational associations between low serum 25(OH)D and adverse aging outcomes, it is important to consider the potential for residual confounding and reverse causation. For example, older adults with lower serum 25(OH)D levels tend to spend less time outdoors, which may reflect reduced mobility, sarcopenia, or social isolation rather than inadequate vitamin D intake per se [1,115]. Lower 25(OH)D is also associated with higher BMI, chronic low-grade inflammation, and poorer overall dietary quality, all of which are independently linked to accelerated biological aging and frailty [97]. Furthermore, chronic disease states and acute illness can acutely lower serum 25(OH)D through hemodilution and altered binding protein metabolism, creating a scenario where low 25(OH)D is a consequence rather than a cause of poor health [13,115]. Thus, observational associations should be interpreted with caution: low 25(OH)D may serve as a marker of frailty or ill health rather than a direct causal driver of epigenetic aging. This distinction underscores the need for caution when translating observational findings into supplementation recommendations and highlights the importance of relying primarily on randomized controlled trial evidence for causal inference.

### 6.2. Sources of Interindividual Variability

Interindividual variability in responsiveness to vitamin D is substantial and rooted in both genetic and non-genetic factors. VDR polymorphisms (e.g., *FokI*, *TaqI*, *BsmI*, *ApaI*) and variants in metabolic enzymes (e.g., *GC*, *CYP2R1*, *CYP24A1*) significantly affect circulating 25(OH)D bioavailability, VDR binding affinity, and downstream epigenetic efficacy [116,117]. Sex differences also play a role: female embryos and adult females generally exhibit higher global DNA methylation levels than males, which may modulate VDR-mediated transcriptional outcomes [51]. Moreover, baseline epigenetic status—such as the degree of CpG island hypermethylation at specific gene loci—determines whether vitamin D can exert a detectable effect [27].

### 6.3. The J-Shaped Dose–Response and Tissue-Specific Barriers

The relationship between vitamin D status and health outcomes is often described as J-shaped [118,119]. However, it is critical to emphasize that this non-linear association is characterized for circulating serum 25(OH)D concentrations, not for oral supplementation doses. Supplement dose (IU/day) and serum 25(OH)D concentration (nmol/L) are not interchangeable, as the conversion between them is modulated by BMI, skin pigmentation, latitude, season, adherence, and genetic polymorphisms in genes such as GC, CYP2R1, and CYP24A1 [14,120]. Epidemiological and some interventional data suggest that both low (<50 nmol/L) and very high (>125–150 nmol/L) serum 25(OH)D levels may be associated with increased risks of certain adverse outcomes, including falls, immunosuppression, or hypercalcemia-related events [54,110,121,122]. However, the upper threshold remains debated and is likely outcome-specific [97]. In the context of oral supplementation, many studies have used doses of 800–2000 IU/day to raise serum 25(OH)D concentrations into the 75–125 nmol/L range in a substantial proportion of participants. Most long-term supplementation trials (1–5 years of intervention) with daily doses ≥ 4000 IU/day have reported no major safety signals, but some have observed potential adverse effects in certain subgroups; these findings are not uniform, and the corresponding serum 25(OH)D levels achieved in those trials varied considerably across individuals [121,123]. Crucially, the conversion from supplemental dose to serum 25(OH)D is highly variable and influenced by baseline status, BMI, skin pigmentation, latitude, season, adherence, and genetic polymorphisms in genes such as GC, CYP2R1, and CYP24A1 [124,125]. Therefore, dose titration must be guided by individualized serum 25(OH)D monitoring, rather than a uniform IU/day cutoff. This non-linearity necessitates careful, personalized dose titration and reinforces that high-dose indiscriminate supplementation is not advisable.

Tissue-specific barriers further limit the efficacy of systemic supplementation. For example, the blood–brain barrier restricts vitamin D entry into the central nervous system, where brain concentrations are only 10–30% of serum levels [106].

Additionally, local activation of vitamin D depends on cell-type-specific expression of CYP27B1; neutrophils lack significant CYP27B1 and cannot produce active 1,25(OH)_2_D_3_ locally, whereas macrophages and T cells can [53]. These differences imply that even optimal serum 25(OH)D levels may not guarantee epigenetic benefits across all organs.

The adverse outcomes associated with the upper arm of the J-shaped curve, primarily derived from observational studies, include an increased risk of falls, hypercalciuria, nephrolithiasis, and, in some studies, a paradoxical increase in all-cause mortality [118,119]. However, the population prevalence of these events at serum 25(OH)D levels > 125 nmol/L is not well-established and appears to be low in generally healthy populations. For example, the large VITAL and DO-HEALTH RCTs, which included participants whose serum 25(OH)D levels sometimes reached or exceeded 125–150 nmol/L with supplementation, did not consistently observe an increase in these specific adverse events [30,122]. This discrepancy between observational and trial data may be explained by residual confounding in observational studies, where very high 25(OH)D levels could be a marker of excessive supplementation or underlying disease, rather than a direct cause of harm. Furthermore, the J-shaped relationship is characterized for circulating serum 25(OH)D concentrations, not for oral supplementation doses. The conversion from dose to serum level is highly variable, making it difficult to define a universal toxic threshold. While some studies suggest that sustained serum levels > 150 nmol/L should be avoided, the evidence for harm in the 125–150 nmol/L range is inconsistent and warrants further investigation [97,121].

### 6.4. Clinical Evidence: Stand-Alone vs. Combined Vitamin D Interventions

To clarify the translational value of vitamin D for healthy aging, it is critical to distinguish settings where monotherapy delivers measurable benefits from those where combined strategies yield superior outcomes. Stand-alone vitamin D supplementation demonstrates consistent benefits primarily in populations with confirmed nutritional deficiency (serum 25(OH)D < 50 nmol/L). In this context, correction of deficiency reliably improves bone mineral homeostasis, reduces the risk of osteomalacia, and lowers fall risk in severely deficient older adults [16,85]. However, in community-dwelling older adults with baseline vitamin D sufficiency (>50 nmol/L), large randomized controlled trials such as VITAL and DO-HEALTH consistently show that vitamin D monotherapy does not significantly reduce fracture risk, cardiovascular events, cancer incidence, or cognitive decline [18,96,107], and cannot reverse established aging-associated epigenetic alterations.

Combined intervention strategies yield more robust and consistent epigenetic and clinical benefits. When paired with resistance exercise, vitamin D supplementation significantly improves muscle strength and reduces fall risk compared with exercise alone [85]; mechanistically, exercise upregulates VDR expression and enhances chromatin accessibility, synergizing with VDR-mediated epigenetic programming [12,80,126]. As detailed in Section 6.1, large-scale trials including DO-HEALTH and VITAL have consistently demonstrated that the combination of vitamin D with omega-3 fatty acids and/or exercise yields superior epigenetic and clinical benefits compared with vitamin D alone [30,75]. These findings indicate that vitamin D functions as an adjuvant epigenetic modulator rather than a standalone anti-aging therapy, and its geroprotective potential is best realized within multimodal lifestyle and nutritional regimens.

### 6.5. Future Research Directions

Building on current evidence, future research priorities are stratified into near-term clinically actionable directions and longer-term exploratory concepts to strengthen translational relevance.

#### 6.5.1. Near-Term Clinically Feasible Priorities

These priorities address key limitations of current research and represent near-term actionable avenues with clear pathways to clinical translation:

(a) RCTs of optimized combined regimens: Long-term multicenter randomized trials are needed to verify the synergistic epigenetic and clinical benefits of vitamin D combined with resistance exercise and nutritional interventions, and to define the optimal dose window and combination protocols for populations with different baseline vitamin D status.
(b) Personalized supplementation clinical pathways: Clinical decision tools integrating VDR polymorphisms, baseline serum 25(OH)D concentration, BMI, and comorbidities should be developed to guide individualized dose titration. Serum 25(OH)D monitoring should be promoted to replace one-size-fits-all fixed-dose regimens, avoiding harm from the J-shaped dose–response relationship.
(c) Clinical translation of epigenetic biomarkers: Epigenetic clocks such as PhenoAge and GrimAge should be validated as surrogate endpoints for vitamin D intervention efficacy. Diagnostic criteria for “functional vitamin D deficiency” based on epigenetic markers should be established, distinguishing nutritional insufficiency from impaired epigenetic responsiveness.

#### 6.5.2. Long-Term Exploratory Research Directions

These directions rely on emerging technologies and address fundamental mechanistic barriers, with longer translational timelines:

(a) Tissue-specific delivery and local activation: Novel vitamin D analogs and targeted delivery systems should be developed to overcome tissue-specific barriers, particularly blood–brain barrier penetration and cell-type-dependent CYP27B1 expression limitations, to improve epigenetic efficacy in the central nervous system and articular cartilage.
(b) Single-cell epigenetic dissection and targeted editing: Single-cell multi-omics technologies (scATAC-seq, scChIP-seq) should be applied to dissect cellular heterogeneity in vitamin D responsiveness across tissues and aging stages [25]. CRISPR-based epigenome editing tools can be used to validate causal roles of VDR-regulated epigenetic loci in aging, though this approach remains highly exploratory.
(c) AI-guided precision nutrition: Artificial intelligence models integrating multi-omics data (genome, epigenome, metabolome) and lifestyle factors should be constructed to accurately predict individual responsiveness to vitamin D supplementation, enabling precision nutritional strategies for healthy aging.

## 7. Conclusions

Vitamin D, through VDR-mediated epigenetic regulation, may help counteract age-related alterations in DNA methylation, histone modifications, chromatin remodeling, and non-coding RNA networks. However, evidence from large randomized controlled trials indicates that vitamin D monotherapy generally provides limited benefits for aging-related outcomes, whereas measurable anti-aging effects have been observed more consistently when vitamin D is combined with exercise and nutritional interventions. This limited efficacy is largely attributable to multiple modulating factors, including baseline vitamin D status, interindividual genetic and physiological variability, a potentially J-shaped dose–response relationship, and tissue-specific barriers to vitamin D signaling. Consequently, supplementation strategies must be individualized: for individuals with serum 25(OH)D concentrations below 50 nmol/L, serum-guided supplementation—typically at 800–2000 IU/day—may be used to achieve target concentrations of approximately 75–125 nmol/L, a range associated with favorable epigenetic and immune effects; for those who are already vitamin D sufficient, indiscriminate supplementation is not supported. In summary, vitamin D should be incorporated into serum-monitored, individually titrated, and multimodal healthy-aging strategies, with supplementation directed primarily toward individuals with documented deficiency and integrated with exercise and other lifestyle interventions.

## Figures and Tables

**Table 1:** Summary of key studies on vitamin D-mediated epigenetic regulation in aging.

Organ System	Epigenetic Mechanism	Key Targets	Author, Year	Evidence Level	Core Findings	Clinical Implication	Ref.
General Mechanisms	Histone modification/chromatin opening	C/EBPα, VDR-bound enhancers	Nurminen et al., 2019	In vitro (human monocyte cell line)	C/EBPα acts as a pioneer factor to promote VDR chromatin binding and H3K27ac deposition	Identifies C/EBPα as a key pioneer factor enabling VDR access to closed chromatin, providing a mechanistic target for modulating myeloid cell epigenetic programming in aging	[9]
General Mechanisms	Genome-wide epigenetic regulation	DNMTs, TETs, VDR binding loci	Nurminen et al., 2018	In vitro (human monocyte cell line)	Vitamin D remodels epigenomic landscape of VDR target genes	Maps genome-wide VDR-dependent epigenetic changes, establishing the global regulatory scope of vitamin D on the human epigenome	[27]
General Mechanisms	3D genome/chromatin remodeling	Enhancer-promoter loops	Warwick et al., 2022	In vitro (human cell line)	VDR activation reshapes spatial genome structure to regulate distal targets	Reveals 3D genome reorganization as a novel mechanism of VDR-mediated distal gene regulation, informing future studies of age-related chromatin architecture decline	[42]
General Mechanisms	Histone methylation (H3K27me3)	*EZH2*, p16INK4a	Yang et al., 2020	In vivo + in vitro (aged mouse bone/cell line)	Vitamin D ameliorates age-related bone loss via the *VDR-Ezh2-p16* axis	Identifies the *VDR-EZH2-p16* axis as a potential target for intervention in age-related bone loss, warranting further translational investigation	[40]
Immune System	Histone modification (H3K27ac)	IL-6, TNF-α, SASP factors	Sayegh et al., 2024	In vitro (human senescent fibroblasts)	Vitamin D suppresses SASP-related inflammatory mediator secretion	Suggests a potential mechanistic basis for vitamin D in attenuating inflammatory pathways; translation to clinical inflammaging requires further study	[52]
Immune System	DNA methylation (*TET2*)	Perforin, granzyme B	Artusa & White, 2025	Preclinical/review	VDR upregulates *TET2* to restore cytotoxic function of aged CD8+ T cells	Provides epigenetic rationale for vitamin D supplementation to preserve adaptive immune function and vaccine response in aging populations	[53]
Immune System	Immune functional regulation	Immune cell subsets	Thouvenot et al., 2025	RCT (clinical isolated syndrome patients)	High-dose vitamin D modulates immune function in demyelinating disease	Provides clinical evidence for high-dose vitamin D as an adjuvant immunomodulatory strategy in neuroinflammatory conditions	[54]
Skeletal Muscle	Histone modification/anabolism	IGF-1, follistatin, PGC-1α	Nurminen et al., 2019; Ryan et al., 2016	In vitro (human skeletal muscle cells)	VDR mediates H3K27ac deposition to regulate muscle anabolism and mitochondria	Elucidates epigenetic mechanisms of vitamin D-regulated muscle protein synthesis and mitochondrial function, informing sarcopenia intervention research	[9,12]
Skeletal Muscle	Myogenic differentiation regulation	*MSTN*, myogenic genes	Braga et al., 2017	In vitro (muscle-derived stem cells)	Vitamin D induces myogenic differentiation and modulates muscle growth regulators	Supports a role for vitamin D in muscle stem cell regulation, with potential implications for age-related muscle mass maintenance	[55]
Skeletal Muscle	Epigenetic clock alteration	PhenoAge, GrimAge2	Bischoff-Ferrari et al., 2020	RCT (combined intervention, older adults)	Vitamin D plus exercise slows epigenetic aging over 3 years	Demonstrates that multimodal intervention including vitamin D can decelerate biological aging markers, supporting translational value of combined lifestyle-nutritional strategies	[30]
Bone & Cartilage	Histone modification (H3K27ac)	*RANKL*	Yang et al., 2020; Zhang et al., 2023	In vivo/preclinical (aged rodent bone)	VDR inhibits aberrant RANKL enhancer activation to suppress excessive osteoclastogenesis	Reveals epigenetic mechanism of vitamin D-mediated osteoclast regulation, providing targets for osteoporosis prevention in aging	[40,56]
Bone & Cartilage	DNA methylation/Wnt pathway	*DKK1*, *SOST*, *RUNX2*	Wimalawansa, 2012	In vitro/review (stem cells/chondrocytes)	Vitamin D regulates Wnt pathway methylation to maintain bone-cartilage homeostasis	Supports epigenetic modulation of Wnt signaling as a mechanism by which vitamin D preserves bone and cartilage integrity with age	[57]
Nervous System	DNA methylation	*APP, BACE1, MAPT*	Skv et al., 2024	In vitro + in vivo (neurodegeneration models, review)	VDR recruits DNMTs to increase methylation of amyloid and tau genes	Identifies DNA methylation of neurodegeneration-related genes as a potential mechanism of vitamin D-related neuroprotection in Alzheimer’s disease	[58]
Nervous System	Calcium homeostasis/protein aggregation	*SNCA*, calbindin-D28k	Rcom-H’cheo-Gauthier et al., 2017	In vitro (neuroblastoma cell line)	Vitamin D inhibits α-synuclein aggregation via calcium regulation	Provides mechanistic support for investigating vitamin D as a disease-modifying agent in Parkinson’s disease	[59]
Nervous System	Neurotrophic and cholinergic regulation	*BDNF*, α7 nicotinic receptor	Manjari et al., 2023	In vivo (Huntington’s disease mouse model)	Vitamin D alleviates motor and cognitive dysfunction in neurodegenerative model	Supports preclinical evidence for vitamin D-mediated neuroprotection in Huntington’s disease, warranting further translational investigation	[60]

Notes: Study design definitions: In vitro = cell culture-based experimental study; In vivo = animal model-based experimental study; Observational = human population association study; RCT = randomized controlled trial; Review = narrative or systematic review synthesizing preclinical and clinical evidence. Findings derived from disease models (e.g., osteoporosis, neurodegenerative disorders) reflect pathological states and cannot be directly extrapolated to physiological aging. All reference numbers correspond to the full reference list at the end of the manuscript. The table compiles representative core studies discussed in the review, covering major epigenetic regulatory mechanisms across each organ system.

## Data Availability

No new data were created or analyzed in this study. Data sharing is not applicable to this article.

## Abbreviations

The following abbreviations are used in this manuscript:

1,25(OH)_2_D_3_	1,25-dihydroxyvitamin D_3_
25(OH)D	25-hydroxyvitamin D
ALS	Amyotrophic lateral sclerosis
APP	Amyloid precursor protein
ATAC-seq	Assay for Transposase-Accessible Chromatin using sequencing
BACE1	Beta-site amyloid precursor protein cleaving enzyme 1
BDNF	Brain-derived neurotrophic factor
BGLAP	Bone gamma-carboxyglutamate protein (osteocalcin)
C/EBPα	CCAAT/enhancer-binding protein alpha
ChIA-PET	Chromatin Interaction Analysis by Paired-End Tag Sequencing
ChIP-seq	Chromatin Immunoprecipitation Sequencing
circRNA	Circular RNA
COL2A1	Collagen type II alpha 1 chain
CYP27B1	Cytochrome P450 family 27 subfamily B member 1
DKK1	Dickkopf WNT signaling pathway inhibitor 1
DNMT	DNA methyltransferase
DO-HEALTH	Vitamin D Supplementation, Omega-3 Fatty Acid Supplementation, and Strength-Training Exercise Program trial
DR3	Direct repeat with 3-base-pair spacing
EZH2	Enhancer of zeste homolog 2
GDNF	Glial cell line-derived neurotrophic factor
HAT	Histone acetyltransferase
HDAC	Histone deacetylase
HD	Huntington’s disease
Hi-C	High-throughput chromosome conformation capture
HSC	Hematopoietic stem cell
IGF-1	Insulin-like growth factor 1
IU	International unit
KDM6B	Lysine demethylase 6B
LINE-1	Long interspersed nuclear element 1
lncRNA	Long non-coding RNA
MAPT	Microtubule-associated protein tau
miRNA	MicroRNA
MSTN	Myostatin
NGF	Nerve growth factor
PD	Parkinson’s disease
PGC-1α	Peroxisome proliferator-activated receptor gamma coactivator 1-alpha
PRC2	Polycomb repressive complex 2
PU.1	Transcription factor PU.1 (SPI1)
RANKL	Receptor activator of nuclear factor kappa-B ligand
RCT	Randomized controlled trial
RUNX2	Runt-related transcription factor 2
RXR	Retinoid X receptor
SASP	Senescence-associated secretory phenotype
SETD7	SET domain containing 7 (histone lysine methyltransferase)
SFRP1	Secreted frizzled-related protein 1
SOX9	SRY-box transcription factor 9
SOST	Sclerostin
SP7	Sp7 transcription factor (osterix)
SWI/SNF	Switch/sucrose non-fermentable
TAD	Topologically associating domain
TET	Ten-eleven translocation methylcytosine dioxygenase
TNF-α	Tumor necrosis factor alpha
VDR	Vitamin D receptor
VDRE	Vitamin D response element
VITAL	VITamin D and OmegA-3 TriaL
Wnt	Wingless-type MMTV integration site family
